# Bidirectional Regulation of NLRP3 Inflammasome and Mitochondrial Quality Control in Sepsis: Mechanisms and Therapeutic Implications

**DOI:** 10.1155/mi/3168669

**Published:** 2026-01-28

**Authors:** Xiangxin Liao, Yixun Wang, Zhaohui Zhang, Xingguang Qu, Gaosheng Zhou

**Affiliations:** ^1^ Department of Critical Care Medicine, The First Clinical Medical College of China Three Gorges University, Yichang, China; ^2^ Department of Critical Care Medicine, Yichang Central People’s Hospital, Yichang, China, yc-hospital.com.cn; ^3^ Yi-chang Sepsis Clinical Research Center, Yichang, China; ^4^ Hubei Provincial Clinical Research Center for Critical Care Medicine (Sepsis Research Collaborative Unit), Yichang, China; ^5^ Medical Administration Department, Yichang Central People’s Hospital, Yichang, China, yc-hospital.com.cn

**Keywords:** mitochondrial dysfunction, mitochondrial quality control, NLRP3 inflammasome, sepsis, targets, treatment

## Abstract

Characterized by its capacity to induce organ failure, sepsis constitutes a life‐threatening pathological state with high incidence and mortality rates. Current treatments primarily focus on antimicrobial therapy and organ support, lacking direct interventions targeting the restoration of cellular or organelle function. Among these mechanisms, mitochondrial dysfunction and overactivation of the NLR family pyrin domain‐containing 3 (NLRP3) inflammasome stand out as key pathological hallmarks. As a classic inflammasome, the NLRP3 inflammasome, upon activation, drives cellular pyroptosis and massive release of inflammatory mediators. Beyond their role as cellular energy generators, mitochondria participate in the modulation of inflammatory responses and oxidative stress control. Mitochondrial quality control (MQC) serves as a prerequisite for the orderly performance of mitochondrial physiological functions. Disruption of MQC invariably results in mitochondrial dysfunction, triggering liberation of mitochondrial reactive oxygen species (mtROS) along with mitochondrial damage‐associated molecular patterns (mtDAMPs), which serve as direct triggers for NLRP3 inflammasome formation and stimulation. This process disrupts MQC, exacerbates mitochondrial dysfunction, and forms a mutually reinforcing “MQC imbalance‐NLRP3 overactivation” vicious cycle that drives disease progression. This review aims to: (1) systematically elucidate the complex bidirectional regulatory mechanisms between the NLRP3 inflammasome and MQC in the context of sepsis, (2) summarize the latest research progress on targeted intervention strategies based on this vicious cycle, and (3) discuss the challenges in clinical translation and future directions of these strategies.

## 1. Introduction

Sepsis represents a complex clinical syndrome resulting from infection, involving physiological, pathological, and biochemical disturbances, and continues to be one of the major global causes of illness and death [1, 2]. Its diverse clinical manifestations hinder timely diagnosis and treatment [3]. In 2017, there were ~48.9 million cases of sepsis worldwide, with ~11 million deaths, accounting for nearly 20% of total global deaths [4]. Sepsis development encompasses intricate pathophysiological mechanisms, including inflammatory responses, defective mitochondrial function, impaired immunity, coagulation abnormalities, neuroendocrine‐immune system disturbances, endoplasmic reticulum stress, and autophagic dysregulation, which together drive the progression toward organ dysfunction [5]. In sepsis, the activation of the NLR family pyrin domain‐containing 3 (NLRP3) inflammasome promotes the massive secretion of pro‐inflammatory cytokines, thereby amplifying the inflammatory cascade and contributing to tissue injury and multi‐organ dysfunction [6].

As part of the nucleotide‐binding oligomerization domain (NOD)‐like receptor family, NLRP3 functions as an intracellular pattern recognition receptor that critically orchestrates the innate immune system’s defensive mechanisms [7]. Activation of the NLRP3 inflammasome is triggered by multiple distinct stimuli [8]. Current evidence indicates that mechanisms, including mitochondrial dysfunction, ionic imbalance, reactive oxygen species (ROS) generation, and disruption of lysosomal integrity, promote its assembly and activation through synergistic or independent pathways [8]. The activation of the NLRP3 inflammasome involves two distinct signals: the first, known as the priming signal, enhances NLRP3 expression through the Toll‐like receptor (TLR)/nuclear factor‐κB (NF‐κB) pathway; the second arises from diverse pathogen‐associated molecular patterns (PAMPs) and damage‐associated molecular patterns (DAMPs), ultimately resulting in inflammasome assembly and its activation [9]. NLRP3 contains three unique domains: a C‐terminal leucine‐rich repeat (LRR) domain responsible for regulatory roles; an NAIP, CIITA, HET‐E, and TP1 (NACHT) domain that facilitates protein oligomerization; and an N‐terminal pyrin (PYD) domain essential for transmitting downstream signals [10, 11]. The PYD typically mediates homotypic interactions to regulate downstream signaling; the LRR primarily facilitates protein–protein interactions; and the NACHT domain exhibits nucleotide‐binding capacity and ATP hydrolysis activity [12]. Once activated, NLRP3 associates with the adaptor protein apoptosis‐associated speck‐like protein containing a CARD (ASC), which then engages pro‐caspase‐1 through homotypic interactions involving PYD–PYD and CARD–CARD domains, respectively [13]. The effector protein caspase‐1 undergoes self‐cleavage and activation, which in turn mediates the cleavage of pro‐interleukin‐1β (pro‐IL‐1β) and pro‐interleukin‐18 (pro‐IL‐18), as well as the secretion of mature IL‐1β and IL‐18 [14]. Caspase‐1 activation also triggers gasdermin D (GSDMD) cleavage, thereby inducing pyroptosis, a form of programed cell death [15, 16]. Different from the previous classical activation pathway, recent studies have proposed a “Two activation signals” model for NLRP3 activation. On one hand, it relies on the inhibition of oxidative phosphorylation and reduced ATP production; on the other hand, it is associated with nonmitochondrial signals, such as Yoda‐1, gardiquimod, or resiquimod [17, 18].

In eukaryotic cells, mitochondria serve as the main locations where energy metabolism occurs, responsible for maintaining cellular bioenergetics through ATP production [19]. As key immunoregulatory organelles, mitochondria influence innate and adaptive immune processes [20]. Mitochondrial dysfunction is linked to metabolic disorders, energy production deficits, and oxidative stress [21, 22]. Impaired oxygen delivery to mitochondria is a key cause of tissue or organ dysfunction [23]. Evolution has endowed cells with intricate mitochondrial quality control (MQC) mechanisms that coordinate mitochondrial biogenesis, dynamics, and mitophagy to address physiological demands and external perturbations [24]. Upon external stimulation, mitochondria can alter their morphology via fission or fusion; in extreme cases, they are degraded through mitophagy [25]. MQC maintains cellular energy homeostasis by regulating mitochondrial components, products, and byproducts, with mitophagy playing a crucial role in mitochondrial renewal [26]. Abnormalities in MQC are associated with multiple disease entities, including infectious diseases, metabolic disorders, cardiovascular diseases, neurodegenerative diseases, and tumors [27].

In sepsis, a close bidirectional interaction exists between MQC imbalance and NLRP3 overactivation, collectively forming a self‐amplifying vicious cycle that represents the core mechanism underlying organ damage. This review will delve into the regulatory mechanisms of the MQC–NLRP3 axis and explore the therapeutic potential of targeting this axis.

## 2. The Interaction Between NLRP3 Inflammasome Activation and Mitochondrial Damage

### 2.1. Mitochondrial Dysfunction and Mitochondrial Damage‐Associated Molecular Patterns (mtDAMPs) in Sepsis

Upon mitochondrial damage, mtDAMPs, which include mitochondrial transcription factor A (TFAM), mitochondrial DNA (mtDNA), N‐formylmethionyl peptides, cardiolipin, cytochrome c (cyt c), and succinate, are released [28]. These signals can activate innate immune responses by being recognized by pattern recognition receptors and triggering inflammatory reactions [29]. Released mtDAMPs activate multiple signaling pathways in tissue‐resident and immune cells, inducing a series of downstream effects such as ROS production, proinflammatory cytokine generation, degranulation, cytoskeletal rearrangement, and neutrophil extracellular trap (NET) formation [30]. These processes collectively influence the occurrence, morbidity, and mortality of sepsis.

### 2.2. The Interrelationship Among the NLRP3 Inflammasome, Mitochondrial Dysfunction, and MQC

#### 2.2.1. NLRP3 Inflammasome and Mitochondrial Dysfunction

Disrupted cellular activities, such as mitochondrial dysfunction and changes in cell death pathways, have also been shown to influence the development of organ dysfunction [31]. The regulation of mitochondrial impairment in sepsis involves various elements such as MQC, reactive nitrogen species (RNS)/ROS, calcium balance, and alterations in mitochondrial membrane permeability [5, 32–34].

During sepsis, inflamed cells produce large quantities of ROS and RNS [35]. Under physiological conditions, ROS maintain intracellular redox balance and participate in cellular signaling [36]. However, under pathological conditions such as sepsis, excessive production of mitochondrial ROS (mtROS) leads to oxidative stress, which in turn triggers inflammatory responses. Mitochondria in immune and nonimmune cells increase ROS production in response to PAMPs and DAMPs during infection. Sepsis triggers the release of PAMPs and DAMPs. PAMPs originate from invading pathogens such as bacteria, viruses, or fungi, including components like LPS, peptidoglycan, lipoteichoic acid, and bacterial or viral nucleic acids [37]. DAMPs are endogenous molecules released from damaged host cells, such as mtDNA, ATP, high‐mobility group box 1, heat shock proteins, and mtROS [38]. These molecules can be recognized by TLRs, which then act on the MyD88‐NF‐κB pathway. LPS‐induced metabolic reprograming in macrophages generates mtROS through the reverse electron transport pathway of mitochondrial electron transport chain complex I, thereby regulating the release of IL‐1β during NLRP3 inflammasome activation [39]. Elevated ROS levels damage mitochondrial proteins and DNA, and in sepsis patients, mitochondrial impairment and dysfunction further drive excessive ROS production [40], which activates the NLRP3 inflammasome via multiple mechanisms [41, 42]. The interaction between superoxide anion radicals and nitric oxide (NO) results in the formation of peroxynitrite, thereby increasing the levels of RNS [43]. NO is one of the necessary triggers for mitochondrial damage [44]. Related studies have shown that mtROS can activate NLRP3 by inducing mtDNA oxidation [45], and cytosolic oxidized mtDNA is essential for NLRP3 inflammasome activation [46]. Damage to mitochondria causes mtDNA escape into the cytosol, exacerbating lipopolysaccharide (LPS)‐induced cytotoxicity by activating the cyclic GMP‐AMP synthase (cGAS)‐stimulator of interferon genes (STING)‐NLRP3 axis [47].

Calcium ions (Ca^2+^) are key intracellular second messengers that coordinate various cellular functions [48, 49]. Ca^2+^ overload leads to mitochondrial dysfunction, and Ca^2+^‐mediated mitochondrial dysfunction further activates the NLRP3 inflammasome [50–52].

During sepsis, pore formation primarily occurs through three mechanisms: mitochondrial permeability transition (MPT), mitochondrial outer membrane permeabilization (MOMP), and mitochondrial membrane opening mediated by gasdermins [34, 53]. Oxidative stress damaged mitochondria, resulting in reduced mitochondrial membrane potential and triggering of MPT pore, causing the release of mtDNA into the cytoplasmic sol [54]. The phenomenon of MPT involves calcium‐dependent enhancement of mitochondrial membrane permeability, causing mitochondrial enlargement and outer membrane disintegration [55, 56]. MPT and NLRP3 inflammasome activation exhibit a mechanistic interconnection [57].

Mitochondrial dysfunction (such as MOMP or MPT) can cause mtDNA and ROS entering the cytoplasmic compartment, directly activating the NLRP3 inflammasome and triggering IL‐1β/IL‐18 maturation [58]. Imbalance in MQC, especially when there is a defect in mitophagy, results in the accumulation of oxidatively modified mtDNA, exacerbating inflammatory signals. Moreover, NLRP3 activation further promotes mitochondrial ROS production and mtDNA release, forming a malignant positive feedback loop. The relationship between MQC and NLRP3 will be discussed in detail below.

Sepsis contributes to a series of systemic consequences, including systemic vascular endothelial injury, tissue damage, systemic respiratory failure, glutathione depletion, mitochondrial dysfunction, and reduced ATP and O_2_ consumption levels [59]. Therefore, understanding how MQC responds to and mitigates mitochondrial dysfunction is essential.

#### 2.2.2. Mitochondrial Dysfunction and MQC

It is worth noting that survivors of sepsis‐induced multiple organ dysfunction exhibit enhanced mitochondrial biogenesis, which is beneficial for maintaining mitochondrial function and energy status [60]. Experimental studies demonstrated that in murine models of sepsis, the administration of high‐dose hydrogen gas via inhalation could upregulate key mediators participating in mitochondrial biogenesis processes while simultaneously suppressing the expression of dynamin‐related protein 1 (Drp1), which ultimately led to improved mitochondrial functional capacity [61].

Through fusion, mitochondria can share contents such as mtDNA, proteins, and metabolites. Suppressing mitochondrial fusion causes the organelles to fragment, which subsequently contributes to mitochondrial dysfunction [62]. Mitochondrial fusion and fission are core mechanisms maintaining mitochondrial function, and their fine‐tuned regulation is critical for cellular energy metabolism, quality control, and stress responses [63].

Selective mitophagy occurs as a quality control mechanism, precisely recognizing and degrading damaged mitochondria. Heme oxygenase‐1 (HO‐1) induction helps facilitate mitochondrial biogenesis, maintain the dynamic balance of mitochondrial fusion and fission, and enhances the expression of critical mitophagy regulators at both transcriptional and translational levels, thus improving mitochondrial function [64].

#### 2.2.3. Coordinated Regulation Among Key Processes in MQC

The total mitochondrial content in cells is a dynamic balance between mitochondrial biogenesis and mitochondrial degradation (including mitophagy) [65]. Additionally, mitochondrial dynamics are crucial for regulating quantity, subcellular distribution, morphology, and function [66]. These processes collectively maintain mitochondrial homeostasis and modulate mitochondrial form, volume, and function. Currently, the concept of “Mitochondrial Flush” has been proposed, which is a dual‐intervention strategy that restores mitochondrial homeostasis by selectively eliminating damaged mitochondria and synchronously promoting mitochondrial biogenesis [67]. Studies have shown that VB enhances mitochondrial biogenesis in cardiomyocytes by increasing mitophagy and fusion while reducing mitochondrial fission [68]. Meanwhile, mitochondrial biogenesis coordinates with dynamic processes (fusion and fission) to jointly maintain the dynamic balance of mitochondrial quantity and morphology, thereby affecting their spatial distribution and functional status [69]. In sepsis, abnormal fission impairs the mechanism by which mitophagy recognizes damaged mitochondria, thereby exacerbating mitochondrial dysfunction and inflammatory responses [34].

In Figure 1, under the inflammatory storm of sepsis, the NLRP3 inflammasome is activated and assembled in response to various endogenous and exogenous signals. The activated inflammasome disrupts normal cellular activities in sepsis primarily through two key pathways: one is the pyroptosis pathway, and the other is the release of inflammatory mediators. In sepsis, multiple organelles suffer damage to varying degrees. This figure specifically highlights the pathological interplay among the NLRP3 inflammasome, MQC, and mitochondrial dysfunction. Ultimately, their interaction leads to impaired mitochondrial metabolism and reduced energy production.

Figure 1The relationship between NLRP3 inflammasome, mitochondrial quality control, and mitochondrial dysfunction. (a) It briefly summarizes the interactions among the NLRP3 inflammasome, mitochondrial quality control (including biogenesis, fission, fusion, and mitophagy), and mitochondrial dysfunction. (b) Sepsis induces the release of PAMPs and DAMPs, which triggers the TLR‐MyD88‐NF‐κB pathway and mitochondrial dysfunction pathway, ultimately activating the NLRP3 inflammasome and driving pyroptosis and inflammatory responses, while also illustrating the role of mitochondrial quality control in this process.

**Figure:**
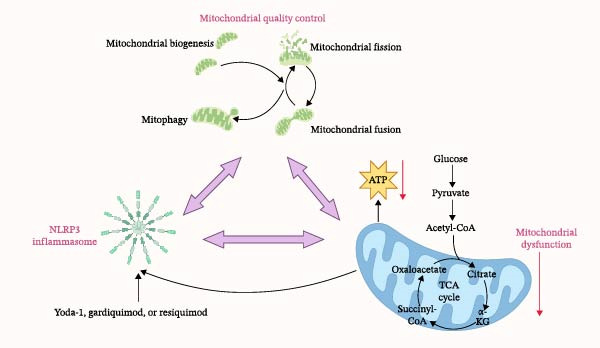
(a)

**Figure:**
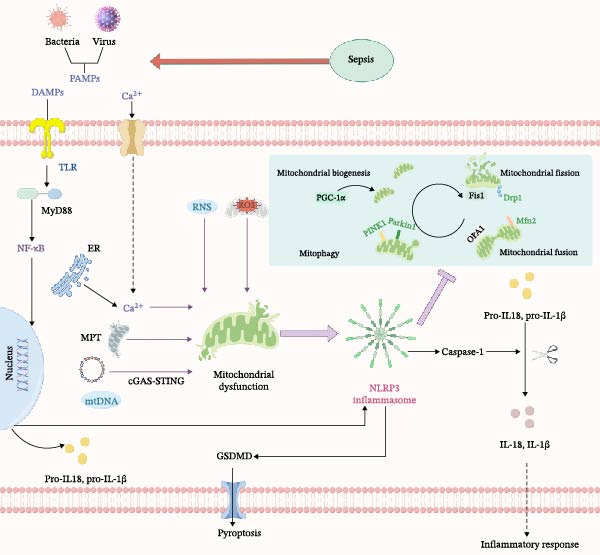
(b)

## 3. The Relationship Between NLRP3 Inflammasome and MQC in Sepsis

### 3.1. NLRP3 Inflammasome and Mitochondrial Biogenesis

Mitochondrial biogenesis describes the formation of new mitochondria originating from existing ones, a process that encompasses mtDNA transcription and translation, along with the synthesis, import, and assembly of mitochondrial proteins encoded by nuclear DNA [70]. This process is crucial for maintaining mitochondrial quality and function. In mitochondrial biogenesis, peroxisome proliferator‐activated receptor gamma coactivator 1α (PGC‐1α) and nuclear respiratory factor 1/2 (Nrf1/2) act as key coactivators and transcription factors, respectively [5]. TFAM, an important regulatory factor between mitochondria and the nucleus, is expressed in the nucleus but functions in mitochondria as a key transcriptional and packaging factor for mtDNA [71, 72]. PGC‐1α is considered a major regulator of mitochondrial biogenesis. Research findings indicate that activation of PGC‐1α can mitigate kidney injury and reduce cellular damage and renal fibrosis by regulating mitochondrial dynamics and the NLRP3 inflammasome pathway [73]. Inhibition of NLRP3 inflammasome activation by malvidin was first demonstrated by Fan et al. [74] with later studies providing evidence for its role in ROS scavenging, mitochondrial membrane potential stabilization, mitochondrial genome replication promotion, and structural integrity maintenance (manifested as attenuated swelling and preserved cristae morphology), and promotes PGC‐1α nuclear translocation, thereby enhancing mitochondrial biogenesis. Furthermore, in this experiment, the use of PGC‐1α inhibitors (SR18292) and Nrf2 inhibitors (ML385) demonstrated that malvidin inhibits NLRP3 inflammasome activation through the PGC‐1α/Nrf2 signaling pathway, thereby alleviating sepsis‐induced acute kidney injury (AKI). AMP‐activated protein kinase (AMPK), as an upstream stimulatory signal for PGC‐1α, can upregulate its expression, regulate mitochondrial biogenesis and fission, reduce ROS production, and alleviate cell apoptosis [75]. Sirtuin 3 (SIRT3) and AMPK stimulate mitochondrial biogenesis. Xin et al. [76] proved that in an LPS model, compared with the control group, the gene transcription levels of PGC1‐α, Nrf2, and TFAM were significantly decreased, and SIRT3 overexpression (via lentiviral SIRT3 delivery) treatment reversed this effect, with AMPK playing an essential role in this process. Apelin suppresses sepsis‐induced myocardial dysfunction by inhibiting NLRP3‐mediated pyroptosis through the activation of the AMPK pathway [77]. Based on the role of AMPK in mitochondrial biogenesis, release of pro‐inflammatory factors IL‐1β and IL‐18 exacerbates inflammatory responses. In severe sepsis, impaired mitochondrial biogenesis leads to increased cytoplasmic mtROS and mtDNA translocation; notably, mtDNA translocation to the cytoplasm is partially dependent on inflammasome activation [78]. Thus, NLRP3 activation further impairs mitochondrial biogenesis.

### 3.2. NLRP3 Inflammasome and Mitochondrial Fusion

Mitochondrial fusion occurs in two modes: one involves the merging of the outer mitochondrial membrane, at the same time, the other pertains to the fusion of the inner mitochondrial membrane. These processes are mediated by the fusion regulators Mitofusin 1 (Mfn1) and Mitofusin 2 (Mfn2), alongside the inner membrane protein Optic Atrophy 1 (OPA1) [79]. Few investigations have been conducted to elucidate the molecular mechanisms through which Mfn1 modulates NLRP3 in sepsis. Through Mfn1‐mediated mitochondrial fusion promotion and Caspase‐11‐dependent NLRP3 inhibition, Xuanfei Baidu Formula (XBF) treatment reduces NF‐κB and MAPK activity, ultimately diminishing proinflammatory macrophage polarization [80]. This suggests that Mfn1 may enhance mitochondrial fusion to inhibit NLRP3 function, thereby reducing inflammatory responses.

Studies have demonstrated that *Mycobacterium tuberculosis* (MTB) upregulates Mfn2 expression and induces IL‐1β secretion; further research has revealed that Mfn2 is involved in NLRP3 inflammasome assembly and activation during MTB infection [81]. Another study indicated that oroxilin A reduces NLRP3 inflammasome activation and apoptotic responses in ethanol‐treated hepatocytes by decreasing mitochondrial ROS via the PGC‐1α/Mfn2 pathway [82]. Mfn2 mediates outer mitochondrial membrane fusion and is involved in the formation of mitochondrial‐associated endoplasmic reticulum membrane (MAM) [83]. MAM, as contact sites between mitochondria and the endoplasmic reticulum, regulates intracellular calcium homeostasis, inflammasome activation, autophagy, and other functions [84]. As a traditional Chinese medicinal agent, *Chimonanthus nitens* Oliv. essential oil (CEO) exhibits dual pharmacological properties: anti‐inflammatory and antioxidant effects [85]. The CEO inhibits Mfn2 expression, thereby suppressing Mfn2‐mediated MAMs formation, blocking NLRP3 inflammasome assembly and activation at the MAMs, reducing NF‐κB p65 phosphorylation, and decreasing the expression of inflammatory factors such as caspase‐1 and IL‐1β, thereby alleviating septic intestinal injury [85]. Interestingly, this contradicts the previous understanding that impaired mitochondrial fusion inhibits NLRP3 inflammasome activation [86], which may be associated with Mfn2 overexpression, highlighting the complexity of Mfn2 function under pathological conditions.

Pyruvate dehydrogenase kinase (PDHK) promotes NLRP3 inflammasome activation, and PDHK inhibitors can reverse this effect [87]. On one hand, mitochondria are involved in NLRP3 inflammasome activation; on the other hand, after LPS + ATP activates NLRP3, the levels of mtROS in macrophages significantly increase, triggering oxidative stress and inducing mitochondrial oxidative damage and dysfunction. Inhibiting PDHK reduces mtROS production; PDHK inhibitor treatment also decreases mitochondrial fission, increases mitochondrial fusion, reduces ATP‐induced Drp1 (Ser616) phosphorylation, and upregulates OPA1 protein levels [87]. This indicates that NLRP3 inflammasome activation promotes mitochondrial fragmentation by increasing mtROS and reducing mitochondrial fusion.

### 3.3. NLRP3 Inflammasome and Mitochondrial Fission

Mitochondrial fission refers to the process by which a mitochondrion divides into two mitochondria, involving the inheritance and distribution of organelles during cell division, the normal distribution of mitochondria, and the release of cyt c during apoptosis [63, 88]. During mitochondrial fission, a cascade of events takes place, with the large GTPase Drp1 being indispensable for proper execution [79]. Drp1 is a cytoplasmic protein that induces mitochondrial fission by interacting with mitochondrial fission protein 1 (Fis1) [89, 90], modulating mitochondrial quality and cellular function through changes in its oligomeric state and various modifications [91]. Drp1 and NLRP3 are involved in various diseases, such as sepsis, AKI, neurodegenerative diseases, acute lung injury (ALI), cognitive impairments associated with aging, and ischemia‐reperfusion injury [92–95], triggering pyroptosis and inflammatory responses. These two proteins interact and synergize, jointly promoting disease progression, and both are potential therapeutic intervention targets. Through the Drp1/ROS/NLRP3 pathway, dynasore attenuates NF‐κB activation in vitro, decreases inflammatory mediator levels, suppresses IL‐1β processing and secretion, while inhibiting NLRP3 inflammasome formation and pyroptosis in macrophages [96].

Mitochondrial dysfunction, along with the buildup of mtROS, mitochondrial DNA (mtDNA), and various mitochondria‐associated proteins and lipids, is critically involved in triggering NLRP3 inflammasome activation [97]. Furthermore, mitochondrial integrity is critically maintained by properly regulated fission and fusion dynamics, and disturbance of this equilibrium results in significant activation of the NLRP3 inflammasome signaling pathway [97]. Mitochondrial fission protein markers (especially Drp1) are positively correlated with oxidative stress and NLRP3‐mediated inflammation [98]. The Drp1‐inhibiting compound schaftoside effectively modulates mitochondrial dynamics by decreasing fission‐promoting protein abundance and increasing fusion‐supporting protein quantities. Notably, schaftoside treatment reduces mtROS accumulation and suppresses NLRP3‐related inflammatory factor mRNA expression. Concurrently, it enhances lung epithelial tight junction protein production and mitigates LPS‐provoked pulmonary histological alterations [98]. Coenzyme Q10 (CoQ10) modulates mitochondrial dynamics while reducing oxidative damage and NLRP3‐mediated inflammatory responses, consequently alleviating LPS‐induced ALI and facilitating reconstruction of alveolar epithelial barrier integrity [98]. Tangeretin (TAN) inhibits the LPS‐induced decrease in Polo‐like kinase 1 (PLK1) expression, reduction in AMPK phosphorylation, and decline in Drp1 (S637) phosphorylation, thereby suppressing excessive mitochondrial fission, reducing ROS production, and further inhibiting NLRP3 inflammasome‐mediated macrophage pyroptosis. Following treatment with the PLK1 inhibitor Volasertib, TAN’s regulatory effect on Drp1 phosphorylation was blocked, NLRP3 inflammasome activation increased, and lung injury worsened, further confirming the important role of the PLK1/AMPK/Drp1 signaling axis in regulating NLRP3 inflammasome activity [99]. ACT001 (DMAMCL), a derivative of Micheliolide, inhibits NLRP3‐mediated apoptosis [100], reduces the production of NETs, and protects cardiovascular function, thereby improving cardiovascular function in sepsis‐induced mice [101]. Mitochondrial division inhibitor 1 (Mdivi‐1) demonstrates protective effects in various disease models by inhibiting Drp1‐mediated mitochondrial fission, such as ameliorating brain injury in septic encephalopathy [102] and regulating M1 macrophage polarization to alleviate atherosclerosis [103]. The research team led by Fan found that pharmacological inhibition of Drp1 using either Mdivi‐1 or ACT001 substantially decreased LPS + ATP‐induced pyroptosis dependent on NLRP3 inflammasome activation, highlighting the therapeutic potential of targeting mitochondrial fission [104]. Both ACT001 and Mdivi‐1 treatments successfully inhibited NLRP3 inflammasome‐mediated IL‐1β secretion. Importantly, ACT001 was shown to mitigate LPS‐triggered ALI by regulating the DRP1/NLRP3 signaling pathway.

The generation of ROS is also crucial for the interaction between Drp1 and NLRP3. Studies have confirmed Nodakenin’s therapeutic effects in reducing osteoarthritic cartilage deterioration and controlling inflammatory reactions in mouse knees, mediated by its regulation of the mitochondrial Drp1/ROS/NLRP3 cascade [105]. Additionally, genetic ablation of Drp1 was found to suppress foam cell generation driven by M1 macrophage polarization through inhibition of the mtROS‐NLRP3 signaling axis [103]. Comparative analysis revealed that while the LPS‐treated septic AKI group exhibited loss of Δψm and heightened mtROS levels, Mdivi‐1 administration partially restored these mitochondrial parameters [106]. Experimental data indicate that downregulating Drp1 effectively limits NLRP3 inflammasome triggering, leading to decreased oxidative stress in kidney tissues during sepsis. Belonging to the somatostatin peptide family, Cortistatin (CST) represents a recently characterized protective factor for cardiovascular health that is expressed throughout the nervous, immune, and endocrine systems, exerting anti‐inflammatory effects and metabolic control [107]. The work of Duan et al. [108] established that CST’s specific affinity for somatostatin receptor 2 (SSTR2) induces AMPK activation, which subsequently inhibits mitochondrial fission mediated by Drp1, reduces ROS accumulation, and attenuates NLRP3‐mediated pyroptosis, ameliorating myocardial injury in sepsis.

The role of Drp1 in neuroprotection and synaptic function has attracted significant attention [109, 110]. In murine models of sepsis‐associated encephalopathy induced by cecal ligation and puncture (CLP), hippocampal neurons exhibited enhanced mitochondrial fission processes, with concurrent upregulation of both phosphorylated Drp1 and NLRP3 inflammasome expression [111]. Decreased levels of NLRP3, cleaved caspase‐1, and GSDMD‐N terminal domains became apparent in BV‐2 cells following Drp1 knockdown via siDrp1 transfection, directly demonstrating the regulatory role of Drp1 in NLRP3 inflammasome activation. The anti‐apoptotic effect of ginsenoside Rg1 on LPS‐challenged human periodontal ligament cells in sepsis is achieved by blocking Drp1‐regulated mitochondrial fission [112]. In the complex pathological state of sepsis, the relationship between Drp1 and the NLRP3 inflammasome exhibits multidimensional interactions, which not only involve alterations in mitochondrial dynamics but also significantly impact oxidative stress levels, inflammatory processes, and cellular function modulation.

By way of CLP, GSDMD becomes activated while Drp1 expression increases, resulting in mitochondrial dysfunction, neuroinflammatory responses, and impairments to neurons and synapses. Importantly, treatment with either necrosulfonamide (NSA) to block GSDMD or Mdivi‐1 to inhibit Drp1 ameliorates these pathological changes, suggesting the GSDMD‐Drp1 axis contributes to sepsis‐associated encephalopathy‐related cognitive dysfunction [113]. Research indicates apolipoprotein E (ApoE) serves as an intrinsic suppressor in murine models of ovalbumin‐provoked allergic airway inflammation. Genetic ablation of ApoE augments NLRP3 inflammasome stimulation, elevating oxidative damage markers (8‐hydroxy‐2′‐deoxyguanosine) and superoxide dismutase 2 levels, with concurrent upregulation of mitochondrial dynamics regulators (OPA1, Mfn2, Drp1, Fis1), implying mitochondrial dysregulation [114]. In diabetic neuropathy, NLRP3 inflammasome activation inhibits the mitochondrial biogenesis pathway via PGC‐1α/Nrf2 and exacerbates mitochondrial fission through Drp1; simultaneously, reduced mitochondrial biogenesis and increased mitochondrial fission further activate the NLRP3 inflammasome [115].

### 3.4. NLRP3 Inflammasome and Mitophagy

Mitophagy has recently emerged as a research hotspot, and this review therefore emphasizes advances in studies on the NLRP3 inflammasome and mitophagy. It is anticipated that further research on mitophagy will significantly contribute to sepsis treatment.

Mitophagy serves as a selective autophagy process that eliminates dysfunctional mitochondria through lysosomal degradation, thereby maintaining MQC and cellular homeostasis [116]. Following mitochondrial injury, depolarization of Δψm occurs alongside enhanced fragmentation, triggering PINK1 accumulation on mitochondrial surfaces that then brings in and turns on Parkin‐a ubiquitin ligase of the E3 type [117, 118]. In patients with sepsis, NLRP3 levels are upregulated, accompanied by downregulation of PINK1 and PARK2 levels, indicating that mitophagy impairment contributes to sepsis pathogenesis [119, 120]. Deficiency in both PINK1 and Parkin potentiates NLRP3 inflammasome stimulation, resulting in elevated release of IL‐1β and IL‐18 cytokines [121].

In sepsis‐induced ALI, SIRT3 deficiency promotes the production of mtROS and the release of mtDNA into the cytoplasm through impaired Parkin‐dependent mitophagy, promoting pyroptosis of pulmonary endothelial cells through the activation of the NLRP3 inflammasome, providing potential therapeutic targets for sepsis‐induced ALI [122]. Silent information regulator 1 (SIRT1) functions as a crucial deacetylase modulating inflammation, mitotic progression, and senescence [123]. Knockout of SIRT1 impairs Rab7‐mediated mitophagic flux at late endosomes, resulting in persistent damaged mitochondria, increased mtROS generation, and cytoplasmic mtDNA release, which hyperactivates NLRP3 inflammasome and the cytoplasmic nucleotide sensing pathway (STING) cascades. The SIRT1–Rab7 regulatory pathway demonstrates protective effects in septic ALI by suppressing NLRP3/STING signaling via late endosomal‐mediated mitochondrial clearance mechanisms [123]. Zn^2+^ can promote mitophagy by inhibiting SIRT7 activity and upregulating Parkin acetylation levels, thereby reducing mtROS production and inhibiting NLRP3 inflammasome activation and pyroptosis‐related proteins [124].

As a crucial transcriptional regulator, Nrf2 mediates cellular defense mechanisms against oxidative damage. It has a complex bidirectional regulatory relationship with mitophagy and jointly participates in mitochondrial mass balance, oxidative stress response, and disease progression. Melatonin treatment mitigates cardiac damage in sepsis through dual mechanisms: stimulation of the Nrf2 pathway cascade and suppression of NLRP3 inflammasome activation [125]. The sesquiterpenoid derivative Micheliolide (MCL) demonstrates both anti‐inflammatory and nephroprotective characteristics. Lei et al. [126] found this compound stimulates mitophagic flux by elevating LC3B, Parkin, and PINK1 expression and reducing P62 accumulation. Additionally, MCL attenuates NLRP3 inflammasome signaling via reduction of NLRP3, caspase‐1, and IL‐1β production [126]. At the same time, when Nrf2 expression was silenced, MCL’s ability to inhibit NLRP3 inflammasome activation was weakened, and its mitophagy‐promoting capacity was impaired. This study demonstrated that MCL promotes mitophagy through the Nrf2/PINK1/parkin axis to inhibit NLRP3 activation, thereby improving LPS‐induced AKI. 4‐Octyl itaconate (4‐OI), chemically derived from itaconic acid and acting on multiple biological targets, exhibits significant anti‐inflammatory activity [127]. In sepsis‐associated AKI, 4‐OI stimulated the Nrf2 pathway, suppressed STAT3 phosphorylation, diminished inflammatory responses and oxidative damage, while promoting mitophagy [127]. This also indirectly demonstrates the effect of mitophagy on NLRP3.

Different chemicals have also demonstrated the relationship between mitophagy and NLRP3 in sepsis. Apelin‐13 (a bioactive peptide) significantly improves survival rates and cognitive function in septic rats while reducing brain injury. Through this therapeutic intervention, PINK1/Parkin‐dependent mitochondrial function was augmented while NLRP3 inflammasome activation was suppressed, consequently diminishing oxidative stress, inflammatory responses, and apoptotic cell death [128]. In vitro, kidney tubular cells were treated with the peptide LTH to enhance binding specificity. Unlike recombinant CD5L at equimolar concentrations, modified CD5L‐loaded fibroblastic reticular cells‐derived exosomes (FRC‐Exos) specifically targeted proximal kidney tubular cells [129]. Their intervention stimulated the Pink1–Parkin mitophagy pathway and antagonized NLRP3 inflammasome activity, thereby attenuating pyroptosis, rehabilitating renal performance, and improving septic survival [129]. Bergapten (BeG) is a furanocoumarin plant hormone found in many herbs and fruits with anti‐inflammatory activity. BeG restored mitochondrial function and attenuated ROS generation following NLRP3 stimulation while upregulating LC3‐II levels, thereby improving LC3‐mitochondria colocalization. Through enhancement of mitophagy and stabilization of mitochondrial dynamics, BeG effectively suppresses NLRP3 inflammasome activation [130]. Prohibitin 1 (PHB1) suppresses NLRP3 inflammasome stimulation through enhancement of mitophagy. Knockdown of PHB1 increased cytoplasmic mtDNA in macrophages by 80% and IL‐1β release by 60%, while the mitophagy inhibitor 3‐MA completely reversed this effect. Mechanistically, PHB1 enhances mitophagy through the PINK1–Parkin pathway, reducing ROS and mtDNA release, thereby blocking inflammasome activation signals [131]. Derived from garlic, the organosulfur compound alliin exhibits antioxidative and anti‐inflammatory characteristics. It suppresses NLRP3 inflammasome stimulation through decreasing intracellular ROS. This compound enhances mitophagy dependent on PINK1/Parkin signaling. When mitophagy was blocked by CsA, alliin’s protective effects against mitochondrial impairment and ROS generation were abolished. In both THP‐1 macrophages and murine mitochondrial preparations, alliin attenuated LPS‐triggered pyroptosis via mitophagy activation [132]. In a rat model of sepsis‐associated AKI, polydatin ameliorated mitochondrial impairment [133]. PD treatment augmented the reduction of mitochondrial mass observed in sepsis, suggesting enhanced mitophagic activity. PD exerted protective effects against mitochondrial dysfunction and apoptotic cell death through inhibition of NLRP3 inflammasome activation, a mechanism dependent on Parkin‐mediated mitophagy [133]. Steroid compound 10 (Compound 10) isolated from *Solidago canadensis* upregulates PINK1, Parkin, and LC3‐II by activating AMPK‐regulated mitophagy, increases the co‐localization of mitochondria and autophagosomes, reduces mtROS production, decreases NLRP3, ASC, and caspase‐1 expression, and reduces IL‐1β/IL‐18 point release [134].

The elevation of cytoplasmic mtDNA in ALI enhances miR‐138–5p promoter methylation, whereas mitophagy‐mediated mtDNA reduction reverses this epigenetic modification, resulting in increased miR‐138‐5p expression that inhibits NLRP3 inflammasome activation and consequently attenuates macrophage pyroptosis and lung inflammatory responses [135]. APPL1 binds to RAB5 to guide early endosomes to damaged mitochondria, promoting p62 recruitment and autophagosome formation, ultimately leading to mitochondrial degradation via lysosomes, reducing mtROS and oxidative mtDNA release, and thereby blocking NLRP3 activation [136].

Mitophagy is the most critical MQC mechanism for suppressing NLRP3 activation. Research by Lin’s group showed that mice lacking NLRP3 or caspase‐1 genes displayed augmented hypoxic responses, mitochondrial redox activity, and mitophagic flux in contrast‐induced kidney injury. This indicates NLRP3 inflammasome inhibition upregulates both hypoxia pathways and MQC mechanisms [137]. Inhibition of NLRP3 inflammasome attenuates apoptosis and upregulates HIF1A and BNIP3‐mediated mitophagy [137]. BNIP3 mediates mitophagy through LC3 interaction [138]. Furthermore, the N‐terminal domain of GSDMD (GSDMD‐NT) interacts with mitochondrial cardiolipin, creating transmembrane pores that disrupt mitochondrial potential, induce mtROS overproduction, and liberate danger‐associated molecules including mtDNA and cyt c [53]. These mitochondrial damage markers subsequently activate mitophagic pathways. Caspase‐3/7/8 cleaves the key autophagy protein Beclin‐1, producing N‐terminal fragments (Beclin‐1‐N) and C‐terminal fragments (Beclin‐1‐C). The cleavage of Beclin‐1 not only directly inactivates the autophagy initiation function but also accompanies the caspase cleavage of PI3KC3, further disrupting the autophagy complex. The recombination of Beclin‐1‐C can also directly induce mitochondrial cyt c release [139]. However, given that few studies have investigated the relationship between caspase‐1 and Beclin‐1, further study of this relationship will help clarify the interrelationship between NLRP3 and mitophagy.

MQC imbalance leads to the release and activation of NLRP3 via mtDAMPs. Defects in different MQC pathways ultimately provoke the buildup and release of mtDAMPs (such as mtDNA) and mtROS, which serve as key mediators directly activating or amplifying NLRP3 signaling. MQC comprises four core physiological processes that maintain a dynamic balance under normal conditions through various regulatory mechanisms. Under septic conditions, MQC exerts a modulatory effect on the NLRP3 inflammasome. Specifically, mitophagy and intact mitochondria act as negative regulators of NLRP3 inflammasome activation, whereas disruption of these processes amplifies inflammasome‐mediated injury. During sepsis, excessive mitochondrial fission and the accumulation of fission‐related proteins or fragments promote the activation of the NLRP3 inflammasome. The mechanism by which mitochondrial fusion influences inflammasome activation remains to be fully elucidated, particularly the potential role of MAM‐localized Mfn2 in facilitating inflammasome assembly.

**Figure 2:**
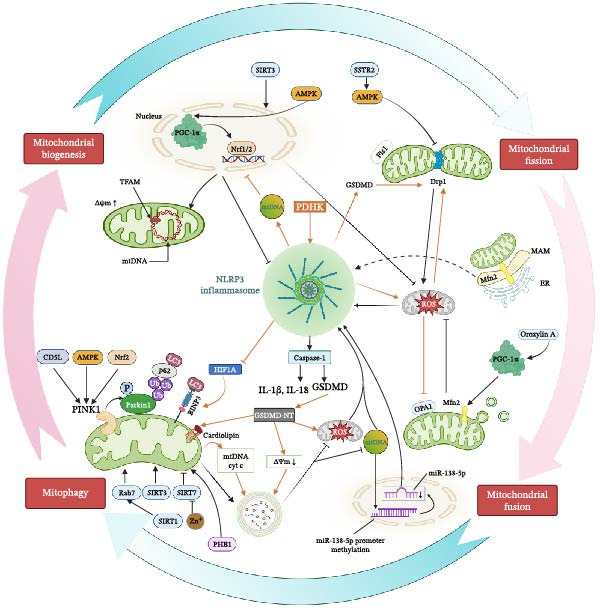
The mutual interaction between the NLRP3 inflammasome and mitochondrial quality control.

As shown in Figure 2, a feedback loop centered on the NLRP3 inflammasome integrates MQC with pyroptotic pathways, inflammatory signaling, and alterations in the cellular microenvironment, forming a vicious cycle characterized by mitochondrial dysfunction and exacerbated inflammation. NLRP3 activation, in turn, further disrupts mitochondrial integrity, perpetuating this cycle. ROS and mtDNA serve as pivotal mediators linking MQC and NLRP3 inflammasome activation.

## 4. Therapeutic Interventions Targeting NLRP3 and Mitochondria in Sepsis

### 4.1. Inhibitors Targeting the NLRP3 Inflammasome and Its Associated Signaling Pathways

#### 4.1.1. Acting on NLRP3 Inflammasome

NLRP3 inflammasome overactivation plays a critical role, triggering an inflammatory cascade that leads to multi‐organ dysfunction [140]. NIMA‐related serine/threonine kinase 7 (NEK7), a crucial serine/threonine kinase of the NIMA family, serves as an essential regulator for NLRP3 inflammasome activation [141]. The C‐terminal portion of NEK7 forms stable interactions with both LRR and NACHT domains in NLRP3, facilitating inflammasome complex formation and stimulation [142]. The selective NLRP3 inhibitor MCC950 exerts its effects by targeting the NACHT domain, disrupting NEK7–NLRP3 binding, preventing ASC oligomerization, and modulating redox homeostasis [143]. In a notable development, MCC950’s Phase II trial for rheumatoid arthritis was terminated because of increased liver enzyme concentrations, including alanine aminotransferase (ALT) and aspartate aminotransferase (AST) [144]. Although its clinical development was hindered by hepatotoxicity, it remains an important tool, drug, and research direction. Currently, various small‐molecule inhibitors targeting NLRP3 have shown promising therapeutic effects in neurological disease models, such as MCC950, CY‐09, and OLT1177 [145]. They effectively inhibit inflammasome assembly and activation by directly suppressing the ATPase activity of NLRP3 or blocking its interaction with NEK7.

The STAT3‐targeting compound ODZ10117 (ODZ) exerts specific inhibition on NLRP3 inflammasome through direct NACHT domain binding. This action interferes with NLRP3–NEK7 association, blocks ASC speck formation, suppresses caspase‐1/IL‐1β activation, and improves outcomes in murine sepsis models induced by LPS [146]. CY09 exerts its inhibitory effects on the NLRP3 inflammasome by binding the ATP site within the NACHT domain, which prevents ATPase activity and oligomer assembly necessary for inflammasome stimulation [147]. In LPS‐induced ALI and sepsis mouse models, exogenous 4‐hydroxynonenal (HNE) or GPX4 inhibition, which increases endogenous HNE, significantly reduced IL‐1β release and tissue inflammatory damage, indicating that HNE is a novel endogenous NLRP3 inflammasome inhibitor [148]. The sesquiterpene lactone ergolide forms irreversible covalent bonds with NLRP3’s NACHT domain, thereby blocking inflammasome complex formation and subsequent activation, which markedly enhances survival outcomes in septic wild‐type mice [149]. Focusing on ASC could represent an alternative therapeutic approach with significant potential. The antitumor agent lonidamine (LND), which inhibits glycolysis, has shown significant anti‐inflammatory properties in experimental studies. LND reduces inflammatory damage in various inflammasome‐related models, such as sepsis and ischemic stroke, by directly binding to ASC and inhibiting its oligomerization [150]. Using an LPS‐triggered septic shock murine model, D359−0396 markedly attenuated inflammatory reactions and organ injury through suppression of the NLRP3–Caspase1–GSDMD pathway, ultimately enhancing survival outcomes [151]. The combination drug Sacubitril/valsartan (SV) protects against LPS‐induced lung injury by dually restraining excessive macrophage activation and inflammatory reactions via targeting NLRP3/GSDMD‐dependent pyroptosis and balancing renin–angiotensin system activity. This mechanism exhibits GSDMD dependance and NLRP3 specificity, providing new drug targets and theoretical basis for the treatment of ALI [152]. Another study by Fu et al. [153] designed and synthesized a series of NLRP3 inflammasome inhibitors based on the dapagliflozin skeleton, among which P33 exhibited significant inhibitory activity. P33 directly binds to the NLRP3 protein, inhibits ASC oligomerization and inflammasome assembly, and has no effect on the NF‐κB activation phase. In vivo experiments showed that P33 significantly improved survival rates and inflammatory markers in LPS‐induced sepsis shock and monosodium urate crystal‐induced peritonitis mouse models, with oral bioavailability reaching 62%, suggesting its promise for treating NLRP3‐associated inflammatory conditions [153]. However, the current application scope and research depth of the aforementioned series of inhibitors are generally inferior to those of MCC950.

As a predominant bioactive constituent in black tea, theaflavin preserves mitochondrial integrity, diminishes mtROS generation, and disrupts NLRP3–NEK7 binding, consequently preventing inflammasome formation and pyroptotic cell death [154]. Tanshinone compounds are the primary active components of Salvia miltiorrhiza, and Tanshinone I inhibits NLRP3 inflammasome formation and subsequent activation through targeted interference with NLRP3‐ASC binding [155]. Dipyridamole, an FDA‐approved drug, inhibits mitochondrial ROS release, directly binds to NEK7 to block its interaction with NLRP3, thereby inhibiting inflammasome assembly [156]. In an LPS‐induced sepsis model, it significantly improved ALI, reduced inflammation, and increased mouse survival rates [156].

Although various direct NLRP3 inhibitors have demonstrated favorable anti‐inflammatory and organ‐protective effects in preclinical models, their clinical translation still faces challenges. Compared with MCC950, novel inhibitors such as ODZ10117 and CY09 block the NLRP3–NEK7 interaction by specifically binding to the NACHT domain, exhibiting higher selectivity, while their long‐term safety remains to be verified.

#### 4.1.2. Action on the Upstream Pathways of the NLRP3 Inflammasome

YL‐109 upregulates CHIP, which acts as an E3 ligase to promote phosphorylation and inhibit NF‐κB activation, thereby suppressing inflammasome activation and alleviating sepsis‐associated inflammatory responses and pyroptosis, exerting protective effects on organs such as the heart, lungs, and intestines [157]. Hederagenin protects against ALI in rats by inhibiting NF‐κB‐regulated NLRP3 inflammasome activation and macrophage M1 polarization, thereby reducing lung inflammation and tissue damage [158]. Ginsenoside Rg1 protects against myocardial dysfunction caused by sepsis. Research indicates that Rg1 mitigates LPS‐induced cardiac dysfunction in mice by decreasing myocardial cell apoptosis and inflammatory factors, primarily through inhibiting the TLR4/NF‐KB/NLRP3 pathway [159]. Through modulation of the TLR4/NOX4/NF‐κB pathway, dexmedetomidine alleviates both NLRP3 inflammasome activation and oxidative stress injury, thereby attenuating LPS‐induced AKI [160]. The antidiabetic drug metformin, commonly used as first‐line therapy for type 2 diabetes, reduces sepsis‐associated ALI in young mice via blockade of S100A8/A9‐TLR4‐NF‐κB signal transduction [161].

Natural products and active components of traditional Chinese medicine, such as berberine (BBR), improve renal function in CLP‐induced SA‐AKI models by inhibiting oxidative stress, protecting mitochondrial integrity (e.g., reducing mitochondrial ROS production, maintaining membrane potential, and blocking TLR4/NF‐κB) [162]. Experimental data reveal sodium tanshinone IIA sulfonate (STS) effectively targets the NLRP3–caspase‐1–GSDMD pathway in both animal and cellular models, exerting neuroprotective effects [163]. LPS‐induced KAT2A binding to α‐tubulin is enhanced, and cichoric acid (CA) disrupts the KAT2A/α‐tubulin complex, reducing acetylated α‐tubulin by 50% [164]. Protein tyrosine phosphatase nonreceptor type 2 (PTPN2) serves as the upstream core molecule through which matrine exerts its effects [165]. Matrine inhibits JNK phosphorylation via PTPN2, thereby reducing SREBP2 maturation and subsequently blocking the interaction between SREBP2 and NLRP3, ultimately suppressing inflammasome activation [165]. The carvacrol exerts cardioprotective effects against LPS‐induced myocardial impairment by suppressing pyroptosis via modulation of the NLRP3–caspase1–GSDMD signaling cascade [166].

Nanomaterials, with their unique physicochemical properties and multi‐target regulatory potential, offer innovative therapeutic strategies for managing inflammatory conditions. To overcome shikonin’s (Shik) poor solubility and toxicological concerns, Guo et al. [167] designed Zn‐Shik‐PEG nanoparticles through a metal‐polyphenol coordination approach. Experimental results demonstrated the nanoparticles’ capacity to scavenge intracellular ROS via Nrf2/HO‐1 pathway activation, while concurrently suppressing NLRP3 inflammasome‐driven inflammatory responses and apoptotic processes through AMPK/SIRT1 signaling modulation [167].

Upstream pathway modulators indirectly inhibit NLRP3 activation by interfering with key signaling nodes such as TLR4/NF‐κB, oxidative stress, and metabolic reprograming, thereby possessing broad‐spectrum anti‐inflammatory and immunomodulatory potential. In general, while upstream interventions avoid the immunosuppressive risks potentially associated with direct NLRP3 targeting, their mechanisms of action are more complex, and their efficacy and safety need to be cautiously evaluated in specific pathological contexts.

#### 4.1.3. Targeting Downstream Pathways of the NLRP3 Inflammasome

Through inhalation delivery of Ac‐YVAD‐CHO, a specific caspase‐1 blocker, endotoxemic rats exhibit diminished production of IL‐18 and IL‐1β in pulmonary and circulatory systems, accompanied by reduced activity of their effector cyclooxygenase‐2 (COX‐2) and enzymes iNOS [168]. Additionally, Ac‐YVAD‐cmk and VX‐765, as caspase‐1 inhibitors, have been shown to reduce mortality and improve cognitive dysfunction in sepsis in mice [169, 170]. As a naturally occurring inhibitor, IL‐18BP counteracts the biological activity of IL‐18. Tadekinin alfa, a recombinant protein of natural IL‐18BP, exhibits high affinity and low immunogenicity. It has completed Phase III clinical trials in hemophagocytic lymphohistiocytosis and demonstrated efficacy in autoimmune diseases, genetic inflammatory diseases, COVID‐19, and cancer [171]. The monoclonal antibody canakinumab, which targets IL‐1β, reduces the risk of cardiovascular adverse events and high‐sensitivity C‐reactive protein levels. However, post‐treatment analysis showed an association between canakinumab and an increased risk of fatal infections [172]. The novel GSDMD inhibitor GI‐Y1 targets the Arg7 residue of GSDMD to inhibit pyroptotic pore formation, thereby blocking GSDMD‐N lipid binding and oligomerization. In vitro studies demonstrated significant reduction of myocardial ischemia and reperfusion injury in sepsis‐induced mouse models, improved mitochondrial function, and no significant toxicity [173]. Disulfiram, an FDA‐approved drug that inhibits pyroptosis by blocking GSDMD pore formation, has been shown to reduce GSDMD‐N activation and lower mtROS levels in sepsis [174, 175].

Downstream intervention strategies focus on the execution phase following inflammasome activation, including caspase‐1 cleavage, IL‐1β/IL‐18 release, and GSDMD‐mediated pyroptosis. Such interventions are characterized by direct action and rapid onset, making them particularly suitable for the stage of severe inflammatory burst that has occurred in sepsis. However, downstream interventions may also interfere with normal immune clearance and tissue repair processes, and long‐term use may increase the risk of infection—for instance, while canakinumab effectively inhibits IL‐1β, it is associated with an elevated risk of fatal infections.

### 4.2. Mitochondria‐Targeted Drugs

The preceding discussion demonstrates the complex relationship between MQC and NLRP3 in sepsis. Therefore, targeted drugs that improve mitochondrial function should, to some extent, alleviate the inflammatory response and pyroptosis induced by NLRP3.

Excessive oxidative stress is a key factor in mitochondrial damage, leading to the production of excessive ROS and RNS that exceed the mitochondria’s clearance capacity, thereby impairing mitochondrial function. This leads to mitochondrial membrane lipid peroxidation and protein damage, thereby impairing normal mitochondrial function. MitoQ maintains mitochondrial citric acid cycle function by upregulating the mRNA and protein levels of citrate synthase in lung tissue and lung macrophages, reducing oxidative stress (lowering mtROS) and apoptosis, thereby alleviating mitochondrial damage [176]. Mitochondria‐targeted vitamin E (MitoVitE) was found to specifically inhibit mitochondrial oxidative stress, enhance mitochondrial antioxidant capacity, reduce hydrogen peroxide production, and protect mitochondrial membrane integrity and respiratory function [177].

Mitochondria‐targeted drugs indirectly inhibit NLRP3 activation through mechanisms such as alleviating oxidative stress, maintaining membrane potential, and promoting energy metabolism, representing a metabolic‐immune integration strategy in sepsis treatment. Although compounds like MitoQ and MitoVitE have demonstrated favorable mitochondrial protective effects in experimental studies, their clinical translation is still limited by issues such as insufficient tissue targeting and unclear pharmacokinetics.

**Figure 3:**
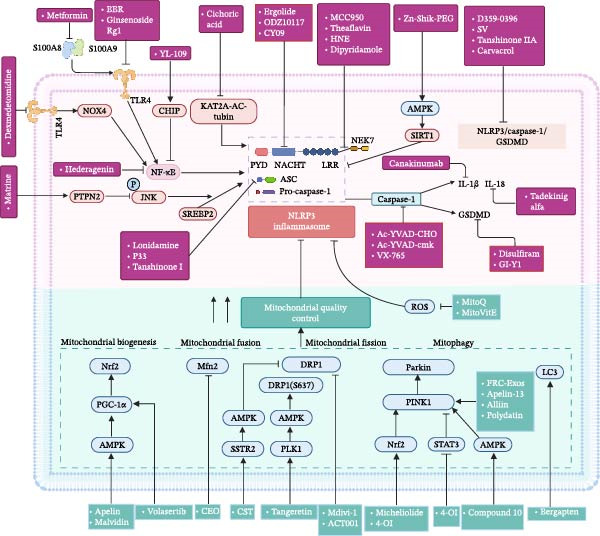
Therapeutic targets and pharmacological agents targeting the NLRP3 inflammasome in sepsis, alongside strategies to restore mitochondrial quality balance.

Based on the aforementioned NLRP3 inflammasome activation and mitochondrial dysfunction, such as fission/fusion abnormalities, mitophagy defects, and mtROS/mtDNA release, Figure 3 summarizes the relevant therapeutic targets and intervention strategies targeting this cross‐regulatory network in current studies.

## 5. Summary and Outlook

MQC imbalance (especially mitophagy defects and excessive fission) leads to mtROS and mtDAMPs release, which is a key mechanism driving NLRP3 inflammasome overactivation; activated NLRP3 further damages MQC through inflammation, pyroptosis, and direct inhibition of mitophagy, forming a self‐amplifying vicious cycle that collectively leads to sepsis‐induced organ injury. Targeting this cycle (inhibiting NLRP3, enhancing MQC such as mitophagy, and regulating key nodes such as AMPK) is a highly promising direction.

Specifically, the details of mtDAMP sensing and signaling remain unclear, such as the preferential binding differences of distinct mtDAMPs to their cognate receptors. Although mtDNA is the most well‐studied mtDAMP, the roles of other mitochondrial‐derived components—including cardiolipin and mitochondrial membrane fragments—in sepsis, as well as their interactive mechanisms with the NLRP3 inflammasome, remain to be fully elucidated. Additional knowledge gaps include: the precise functional role of MAMs in mediating crosstalk between MQC and the NLRP3 inflammasome during sepsis; the organ‐ or cell type‐specific regulatory mechanisms underlying the MQC–NLRP3 axis; and the contributions of noncoding RNAs (circRNAs, lncRNAs) and histone modifications to the modulation of the MQC–NLRP3 axis. Addressing these uncertainties is critical for identifying novel molecular targets to intervene in sepsis‐associated inflammation. Currently, several key challenges hinder the clinical translation of research on the MQC–NLRP3 axis. First, there are no reliable, dynamic biomarkers available to assess MQC status and NLRP3 pathway activity in sepsis patients, which limits the ability to guide precision therapeutic strategies. Second, the clinical relevance of preclinical models—particularly those incorporating clinically relevant factors such as aging or comorbidities—needs further validation to ensure translational applicability. Third, the development of efficient targeted drug delivery systems and the rigorous validation of the safety and selectivity of candidate drugs targeting the MQC–NLRP3 axis remain unmet needs. Collectively, resolving these challenges is essential for advancing research from preclinical studies to viable drug development for sepsis.

To address these challenges, future research should advance from multiple dimensions. Design early‐stage clinical trials targeting the MQC–NLRP3 axis in emergency sepsis patients to assess the impact of drugs on organ function recovery and mortality. Develop organ‐ or cell‐specific targeted delivery systems, such as nanocarriers and antibody/peptide conjugate technologies, to enhance drug targeting and local concentration. In treatment strategies, explore rational combination therapy regimens: for example, combining mitophagy enhancers with low‐dose NLRP3 inhibitors may enhance MQC function while reducing inhibitor toxicity; combining drugs targeting the MQC–NLRP3 axis with immunomodulators to achieve both inflammation control and immune balance. Expand drug development: design structurally optimized inhibitors/activators targeting NLRP3 and MQC regulatory pathways; conduct in‐depth analyses of the precise targets and mechanisms of natural products; and perform structural optimization.

**Nomenclature**
MQC: Mitochondrial quality control
mtROS: mitochondrial reactive oxygen species
mtDAMPs: mitochondrial damage‐associated molecular patterns
NLRP3: NLR family pyrin domain‐containing 3
NOD: nucleotide‐binding oligomerization domain
ROS: reactive oxygen species
TLR: Toll‐like receptor
NF‐κB: nuclear factor‐κB
PAMPs: pathogen‐associated molecular patterns
DAMPs: damage‐associated molecular patterns
LRR: leucine‐rich repeat
NACHT: NAIP, CIITA, HET‐E, and TP1 domain
PYD: pyrin
ASC: apoptosis‐associated speck‐like protein containing a CARD
pro‐IL‐1β: pro‐interleukin‐1β
pro‐IL‐18: pro‐interleukin‐18
GSDMD: gasdermin D
mtDNA: mitochondrial DNA
NET: neutrophil extracellular trap
RNS: reactive nitrogen species
NO: nitric oxide
LPS: lipopolysaccharide
cGAS: cyclic GMP‐AMP synthase
STING: stimulator of interferon genes
Ca^2+^: Calcium ions
MOMP: mitochondrial outer membrane permeabilization
MPT: mitochondrial permeability transition
Drp1: dynamin‐related protein 1
HO‐1: heme oxygenase‐1
PGC‐1α: peroxisome proliferator‐activated receptor gamma coactivator 1α
NRF1/2: nuclear respiratory factors 1 and 2
AMPK: AMP‐activated protein kinase
SIRT3: Sirtuin‐3
Mfn1: Mitofusin 1
Mitofusin: 2Mfn2
OPA1: Optic Atrophy 1
MAM: mitochondrial‐associated endoplasmic reticulum membrane
XBF: Xuanfei Baidu Formula
MTB: *Mycobacterium tuberculosis*
MAMs: mitochondrial‐associated endoplasmic reticulum membranes
CEO: *Chimonanthus nitens* Oliv. essential oil
PDHK: pyruvate dehydrogenase kinase
Fis1: mitochondrial fission protein 1
CoQ10: coenzyme Q10
TAN: Tangeretin
PLK1: Polo‐like kinase 1
Mdivi‐1: mitochondrial division inhibitor 1
CST: cortistatin
SSTR2: aomatostatin receptor 2
CLP: cecal ligation and puncture
NSA: necrosulfonamide
ApoE: apolipoprotein E
Δψm: membrane potential
ALI: acute lung injury
SIRT1: silent information regulator 1
SIRT7: silent information regulator 7
MCL: micheliolide
4‐OI: 4‐octyl itaconate
FRC‐Exos: fibroblastic reticular cells‐derived exosomes
BeG: Bergapten
PHB1: Prohibitin 1
GSDMD‐NT: The N‐terminal domain of GSDMD
Compound 10: steroid compound 10
cyt c: cytochrome c
NEK7: NIMA‐related serine/threonine kinase 7
HNE: 4‐hydroxynonenal
SV: Sacubitril/valsartan
PTPN2: protein tyrosine phosphatase non‐receptor type 2
ALT: alanine Aminotransferase
AST: aspartate Aminotransferase
RAS: renin–angiotensin system
DEX: dexmedetomidine
BBR: berberine
CA: cichoric acid
COX‐2: cyclooxygenase‐2
IL‐18BP: IL‐18 binding protein
MitoVitE: mitochondria‐targeted vitamin E.

## Funding

This work was supported by Beijing Natural Science Foundation (7232126), Special Scientific Research Project of Beijing Critical Care Ultrasound Research Association (2023‐CCUSG‐A‐03), Medical Health Research Project of Yichang (A24‐2‐011), and Health Promotion Project‐Academic construction project of adsorption engineering‐Scientific research and academic promotion project for critical and severe diseases (QS‐XFGCJWZZ‐0049).

## Consent

The authors have nothing to report.

## Conflicts of Interest

The authors declare no conflicts of interest.

## Data Availability

Data sharing not applicable to this article as no datasets were generated or analyzed during the current study.
